# Effects of Adult Exposure to Bisphenol A on Genes Involved in the Physiopathology of Rat Prefrontal Cortex

**DOI:** 10.1371/journal.pone.0073584

**Published:** 2013-09-16

**Authors:** Beatriz Castro, Pilar Sánchez, Jesús M. Torres, Esperanza Ortega

**Affiliations:** 1 Department of Biochemistry and Molecular Biology, Faculty of Medicine, University of Granada, Granada, Spain; 2 Institute of Neurosciences, Faculty of Medicine, University of Granada, Granada, Spain; Universidad Miguel Hernández de Elche, Spain

## Abstract

Several neurological and behavioral dysfunctions have been reported in animals exposed to bisphenol A (BPA). However, little is known about the impact of adult exposure to BPA on brain physiopathology. Here, we focused on prefrontal cortex (PFC) of rats, because it is an important area for cognitive control, complex behaviors and is altered in many psychopathologies. Gamma-aminobutyric acid (GABA) and serotonin (5-HT) systems are essential for PFC function. Therefore, we examined the effects of adult exposure to BPA on 5α-Reductase (5α-R) and cytochrome P450 aromatase (P450arom), enzymes that synthesize GABA_A_ receptor modulators, and tryptophan hydroxylase (Tph), the rate-limiting enzyme in 5-HT biosynthesis. To gain better understanding of BPA’s action in the adult PFC, 84 genes involved in neurotoxicity were also analysed. Adult male and female rats were subcutaneously injected for 4 days with 50 µg/kg/day, the current reference safe dose for BPA. mRNA and protein levels of 5α-R, P450arom and Tph were quantified by real-time RT-PCR and Western blot. Genes linked to neurotoxicity were analyzed by PCR-Array technology. Adult exposure to BPA increased both P450arom and Tph2 expression in PFC of male and female, but decreased 5α-R1 expression in female. Moreover, we identified 17 genes related to PFC functions such as synaptic plasticity and memory, as potential targets of BPA. Our results provided new insights on the molecular mechanisms underlying BPA action in the physiopathology of PFC, but also raise the question about the safety of short-term exposure to it in the adulthood.

## Introduction

In recent years, considerable attention has been focused on endocrine-disrupting compounds and their impacts on the environment and human health, raising questions about their levels of exposure. Bisphenol A (BPA) is an ubiquitous xenoestrogen used in the production of plastic and metal food and beverage containers that can leach into the contents during processing and storage [1]. Moreover, BPA-based resins are commonly used in dentistry [2]. Therefore, most people are exposed almost continuously to BPA in developed countries.

Numerous studies have shown that BPA is able to alter endocrine signalling pathways, leading to adverse biological effects [3]–[6]. Experiments with animals suggest that exposure to this compound may impair brain development, sexual differentiation, cognitive functions and behavior [7]. In addition, several mental disorders, such as schizophrenia, have been linked to BPA [8]. Most research has focused on the neurotoxic effects associated with perinatal exposure to BPA and the mechanism of action behind these neuro-developmental effects [9]. Although recent works point out to adverse effects of BPA on adult brain [10], [11], there is still limited toxicogenomic information on BPA-induced neurotoxicity during adult life.

Central γ-aminobutyric acid (GABA)-ergic transmission plays a key role in controlling emotional state and participates in the regulation of various psychophysiological phenomena [12]. Allopregnanolone (AlloP), the 3α,5α-reduced neurosteroid (3α,5α-NS) of progesterone, is among the most potent known ligands of the γ-aminobutyric acid type A receptor (GABA_A_-R) complex in the central nervous system (CNS) and has anaesthetic, anxiolytic, sedative and anticonvulsant effects, similar to the action of benzodiazepines and barbiturates [13]. The rate-limiting enzyme in the biosynthesis of 3α,5α-NS is steroid 5α-Reductase (5α-R), which is expressed as three isozymes, 5α-R1, 5α-R2 and 5α-R3. While little is known about 5α-R3 function in the brain, the roles of 5α-R1 and 5α-R2 have been largely studied. 5α-R1 is the isozyme mainly implicated in the biosynthesis of 3α,5α-NS [13], and has also a catabolic role, protecting neurons against apoptosis induced by glucocorticoid excess [14]. On the other hand, 5α-R2 might have a masculinizing role in some brain regions of rat, converting testosterone (T) into the more potent androgen dihydrotestosterone [15], [16]. In the brain, T is also converted to estradiol (E2) by cytochrome P450 aromatase (P450arom). Estrogens are able to affect both AlloP levels and GABA_A_-R expression [17].

It has been suggested that GABAergic system may be modulated by the serotonin (5-HT) system. Thus, Waider et al. [18] have recently described that reduction or complete lack of brain 5-HT transmission causes differential changes of GABA systems in prefrontal cortex (PFC), which play an important role in emotional learning and memory processes [19]. Alterations in serotonergic signalling have been also implicated in the pathogenesis of a wide range of neuropsychiatric disorders, including schizophrenia [20], depression [21], impulsive aggression and suicidal behaviour [22]. Tryptophan hydroxylase (Tph) catalyzes the rate-limiting step in 5-HT synthesis and therefore is one of the leading target genes for psychiatric and behavioral disorders [23]. Two isoforms of Tph are known, Tph1 and Tph2. Whereas Tph2 is specifically expressed in the brain, Tph1 is responsible for 5-HT synthesis in peripheral tissues [24].

With this background, the aim of the present study was to evaluate the effects of adult exposure to BPA on 5α-R isozymes, P450arom and Tph isozymes in the PFC of rats, at doses considered safe by the United States Environmental Protection Agency (EPA). In addition, in order to investigate molecular mechanisms of BPA action as a potential neurotoxic agent, we used the PCR Array technology to analyze the expression profile of 84 key genes involved in drug and chemical-induced neurotoxic responses.

## Materials and Methods

### Animals and Treatments

Animals were treated humanely and with regard for alleviation of suffering. All procedures were performed strictly in accordance with recommendations in the Guide for the Care and Use of Laboratory Animals of the National Institutes of Health. Animal welfare and experimental procedures were approved by the Animal Experimentation Ethics Committee of the University of Granada, Spain (Ref. 412-2012-CEEA). Adult male and female Wistar rats weighing 260–280 g and 180–200 g, respectively were housed in stainless steel cages in an air-conditioned room with fluorescent lights on from 7 a.m. to 7 p.m. BPA treatment was randomly distributed across the phases of estrus, because we aimed to design our experiments in a way that reflects the exposure to this endocrine disruptor in real life, where the phase of the menstrual cycle in women is not taken into account when exposition occurs.

Animals were provided with a standard A04 laboratory pellet chow (Panlab, Barcelona, Spain) and water *ad libitum*. Although the concentration of phytoestrogens in the diet was not evaluated, all animals were exposed to the same phytoestrogen levels because the food intake was equivalent for BPA-treated rats and controls. Exposure to endocrine-disrupting chemicals was minimized using glass bottles with rubber stoppers to supply them with tap water. The lab diet provider does not analyze the traceability of BPA in their products. However, considering that the use of plastic in the production, storing, packing and transportation processes is completely avoided, they can almost guarantee the absence of cross contamination with this compound.

Rats were subcutaneously daily injected with 0.2 mL sesame oil containing BPA (Sigma-Aldrich >99% purity) at dose of 50 µg/Kg of body weight (i.e., tolerable daily intake) or with sesame oil alone (controls) for 4 days. Each study group comprised 12 animals. At 30 min after the final injection, rats were euthanized by decapitation, and the brain was removed, frozen in liquid nitrogen, and stored at −80°C until analysis. The dissection of PFC areas was assessed with reference to the Atlas of Paxinos and Watson [25].

The route of BPA administration in the present work is subcutaneous (s.c.) injection while the main route of human exposure is ingestion or through dermal contact with products that leak BPA. According to Batista et al. [6], we used s.c. injection because we need to know exactly the administered doses in order to properly perform mechanistic studies. Moreover, a previous study by Prins et al. [26] stated that despite differences in BPA metabolism, clearance and excretion mechanisms that diverge between rodents and humans and despite differences in BPA pharmacokinetics in route of exposure, the s.c. delivery of BPA employed by these authors provides an internal dose and tissue bioavailability that models internal human levels. Therefore the results presented in this work may be relevant to humans.

### RNA Isolation

Total RNA was extracted from 25 mg of rat PFC tissue with Trizol reagent (Invitrogen), according to the manufacturer’s instructions. RNA samples were then treated with Turbo DNase (Ambion) to remove any contamination with genomic DNA. RNA yield was determined spectrophotometrically by A260/A280 ratio using a NanoDrop ND-1000 spectrophotometer (ThermoFisher). Isolated total RNA integrity was electrophoretically verified by ethidium bromide staining.

### Reverse Transcription and Quantitative Real-Time PCR

First-strand cDNA was synthesized from 1 µg of total RNA following Castro et al. [5]. Absolute quantification of 5α-R1, 5α-R2, 5α-R3, P450arom, Tph1 and Tph2 was performed by real-time PCR using the Techne Quantica™ with SYBR Green PCR Master Mix (Promega). We amplified the target transcripts in parallel with standard curves generated following the method described by Fronhoffs et al. [27]. The amount of mRNA was expressed as number of mRNA copies per micrograms of total RNA.

The PCR profile was as follows: denaturation at 94°C for 30 s, annealing temperature for 30 s, and extension at 72°C for 30 s. The number of cycles was 40 in all cases. At the end of the amplification phase, a melting curve analysis was performed in order to confirm that a single PCR product was detected by the SYBR Green dye. Primers used in the amplifications were designed using Primer 3 software. Primer sequences (5′- 3′), annealing temperatures and GenBank accession numbers for each gene are given in Table 1.

**Table 1:** Primer sequences (5′-3′) and PCR conditions for RT-PCR analysis.

GenBank Acc. No.	Primer	Forward	Reverse	Annealing T^a^ (°C)
NM_017070	**5α-R1**	GAGATATTCAGCTGAGACCC	TTAGTATGTGGGCAGCTTGG	55
NM_022711	**5α-R2**	ATTTGTGTGGCAGAGAGAGG	TTGATTGACTGCCTGGATGG	55
N/A	**5α-R3**	TGCCCATCAGTATAAGTGCC	TCACCATAAAGCTCGAACCAG	50
NM_017085	**P450arom**	TGAGAAGAACGTTCCCTACAG	TCCTCATCTAGATGCAAGGAC	60
NM_001100634	**Tph1**	CCAGCTAGTTCCCAGTCTGC	CTGATTCTCCAGCATCACCAG	63
NM_173839	**Tph2**	CTCCAAGCTTCGCATCACAG	AGCACTTCAGGAAGCGTACC	57

### Neurotoxicity PCR Array

RNA (1 µg) was transcribed to cDNA using the RT^2^ First Strand Synthesis kit (SABiosciences) and analyzed using the Rat Neurotoxicity RT^2^ Profiler™ PCR Array (PARN-096Z; SABiosciences) and the RT^2^ SYBR green PCR master mix (SABiosciences) following the supplier’s protocol. Each array consists of 84 genes known to be involved in drug and chemical-induced neurotoxic responses as well as 12 sequences to control for loading and cDNA quality. For a complete list of genes see http://www.sabiosciences.com/rt_pcr_product/HTML/PARN-096Z.html.

### Electrophoresis and Western Blot Analysis

Protein extraction was performed as previously described [5]. Protein concentration was determined by the dye-binding method of Bradford [28] with BSA as the standard using Bio-Rad protein assay reagent (Bio-Rad Laboratories, Inc, Richmond, CA, USA). Aliquots of samples containing 50 µg of proteins were subjected to 12% sodium dodecyl sulfate-polyacrylamide gel electrophoresis (SDS-PAGE) and western blot following Castro et al. [5]. The blots were incubated overnight at 4°C with primary antibodies at a dilution of 1∶500 for 5α-R1, 1∶400 for Tph-2, 1∶200 for P450arom and 1∶1000 for β-actin, in T-PBS containing 0.5% non-fat dry milk. The blots were incubated for 1 h with the appropriate anti Ig G-horseradish peroxidase (HRP) conjugated secondary antibody at a dilution of 1∶5000. The blots were visualized using enhanced chemiluminescence detection system according to the supplier’s instructions (ECL-Plus, GE Healthcare, USA). The ImageJ program (http://rsb.info.nih.gov/ij/) was used for quantitative analysis of the bands. To account for any differences in loading, target band densitometries were divided by actin densitometries obtained from the same lane. These corrected densitometries were normalized to controls in each experiment.

Antibodies: Goat anti-5α-R1 (Abcam ab110123), rabbit anti-TPH-2 (Thermo Scientific PA1-778), mouse anti-aromatase (ABD serotec MCA2077S). A mouse anti β-actin antibody (Thermo Scientific BA3R) was used as loading control. Goat anti-mouse, goat anti-rabbit and donkey anti-goat Ig G HRP conjugated (Santa Cruz) were used as secondary antibodies.

### Statistical Analysis

Two-way ANOVA, with gender and treatment as independent factors, was used to compare means among multiple groups, applying post hoc pair-wise comparisons with Bonferroni’s penalization, where results were significant. Significance of differences between two groups was determined using Student’s *t*-test. Data are expressed as means ± standard error. A value of p*<*0.05 was considered to be of statistical significance. Statistical and data analysis were conducted using the SigmaPlot for Windows v.11.0 (SPSS Inc., Chicago, IL, USA).

The PCR array data were analyzed using an online analysis tool provided by the supplier (http://www.sabiosciences.com/pcrarraydataanalysis.php). The Ct (threshold-cycle) value was used for calculations of relative amount of mRNA molecules. The Ct value of each target gene was normalized by subtraction of the Ct value from average of five housekeeping genes. This value is defined as the ΔCT. The criteria were a mean difference equal to or greater than 2-fold and *p<*0.05. Ct values greater than 32 were excluded from the data analysis.

## Results

### Effects of BPA on 5α-R Isozymes

**Figure 1:**
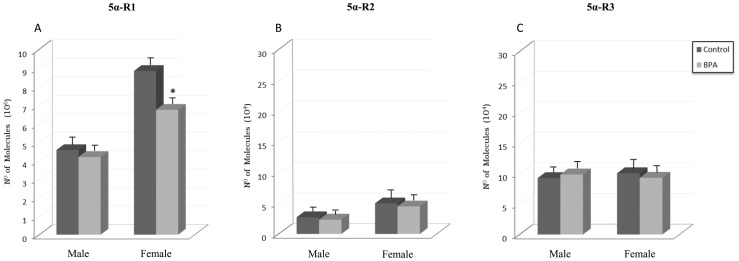
Effects of BPA on mRNA levels of steroid 5α-reductase type 1 (5α-R1) (panel A), 5α-reductase type 2 (5α-R2) (panel B), and 5α-reductase type 3 (5α-R3) (panel C) in prefrontal cortex of BPA-treated male and female rats and their respective controls. * at least p<0.05 vs. their controls.

Figure 1 depicts the mRNA levels of 5α-R1 (panel A), 5α-R2 (panel B) and 5α-R3 (panel C) in PFC of BPA-treated rats and their controls. 5α-R1 mRNA levels were significantly decreased after BPA treatment in females (p<0.005) but not in males in comparison with their respective controls. Two-way ANOVA analyses revealed significant main effects for the BPA treatment [F_(1,28)_ = 10.124, p = 0.004], as well as for the gender [F_(1,28)_ = 81.515, p<0.001] and their interactions [F_(1,28)_ = 5.015, p = 0.033]. No significant differences on 5α-R2 mRNA levels were observed in males and females after BPA administration. Statistical analyses performed with the same design revealed significant main effect for gender [F_(1,28)_ = 41.761, p<0.001] on 5α-R2 mRNA levels; however, no main effects for BPA treatment [F_(1,28)_ = 2.225, p = 0.147] or treatment × gender interaction [F_(1,28)_ = 0.0192, p = 0.891] were identified. No significant differences on 5α-R3 mRNA levels were observed in males and females after BPA administration. No main effects for BPA treatment [F_(1,28)_ = 0.0183, p = 0.893], gender [F_(1,28)_ = 0.0321, p = 0.859], or their interaction [F_(1,28)_ = 0.466, p = 0.500] were found.

**Figure 2:**
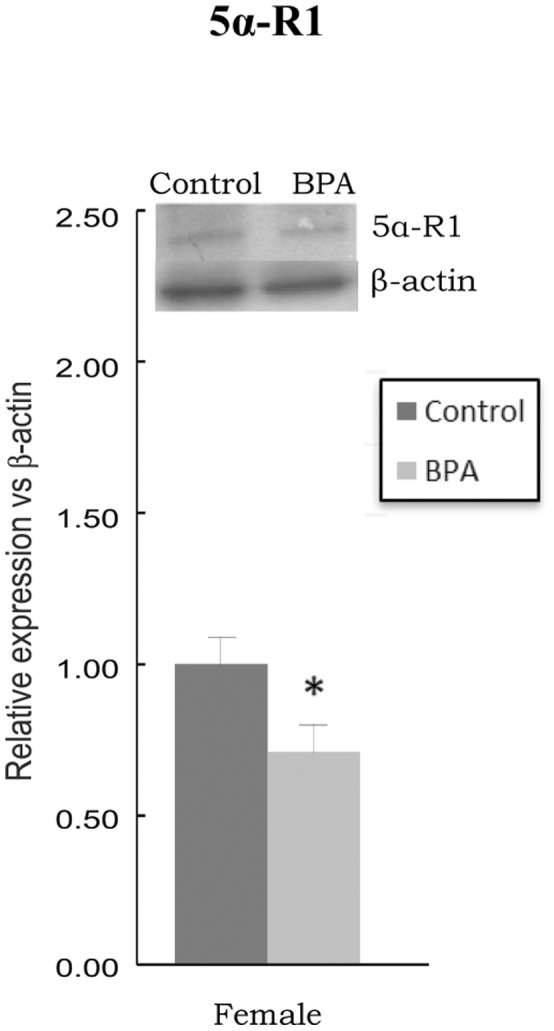
Effects of BPA on protein levels of steroid 5alpha-reductase type 1 (5α-R1) in prefrontal cortex of BPA-treated female rats and controls. * at least p<0.05 vs. their controls.

The protein levels of 5α-R1 were significantly decreased in BPA-treated females versus their controls (p<0.05) (Figure 2). The protein levels were only measured when significant differences in mRNA levels between BPA-treated animals and controls were found.

### Effects of BPA on P450arom

**Figure 3:**
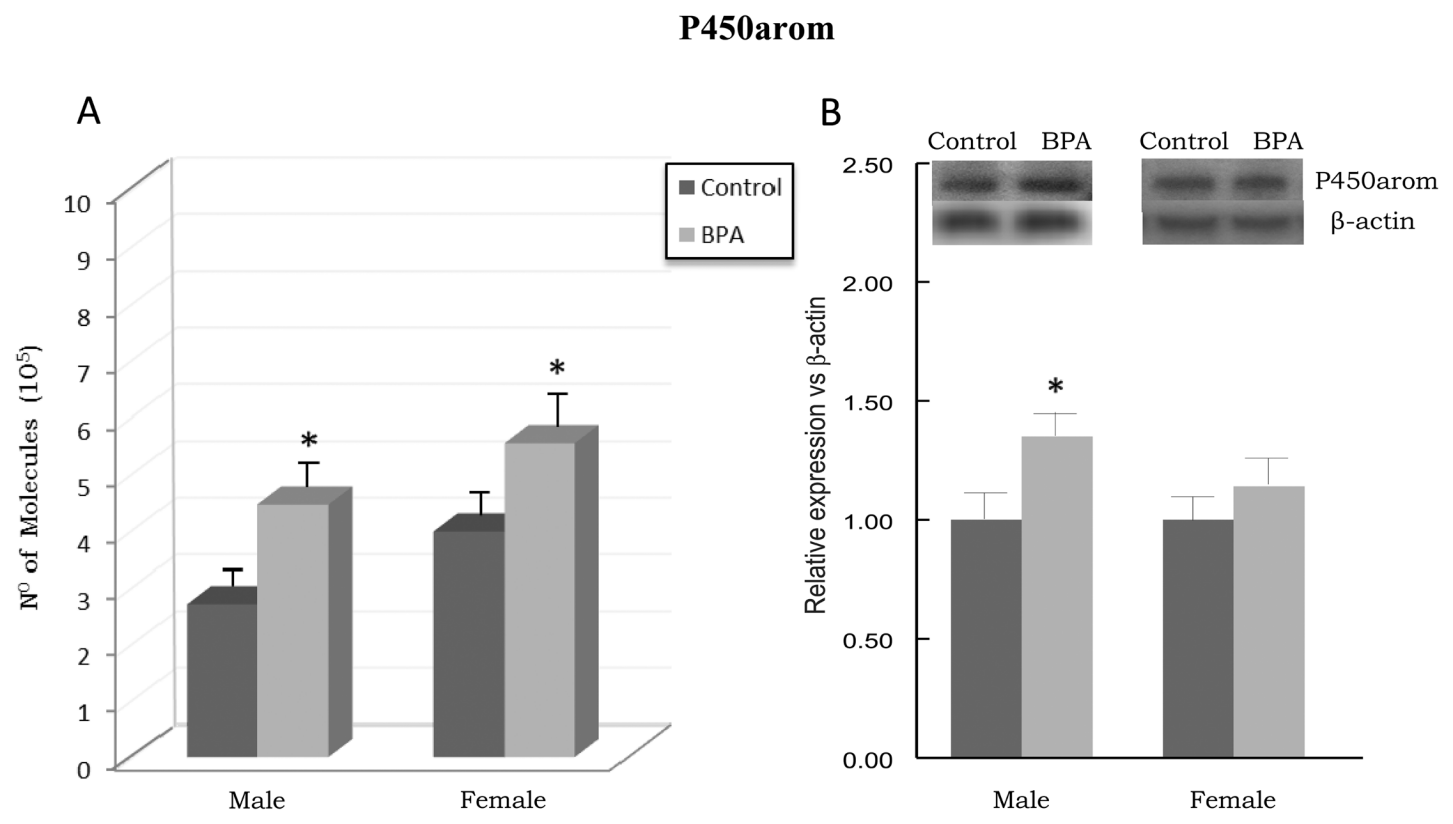
Effects of BPA on cytochrome P450 aromatase (P450arom) mRNA levels (panel A) and protein levels (panel B) in prefrontal cortex of BPA-treated male and female rats and their respective controls. * at least p<0.05 vs. their controls.

Figure 3 depicts the P450arom mRNA levels (panel A) and protein levels (panel B) in PFC of BPA-treated rats and their controls. P450arom mRNA and protein levels were significantly increased in BPA-treated male rats in comparison with their controls (p<0.05). Likewise, P450arom mRNA and protein levels were increased in BPA-treated female rats in comparison with their controls (p<0.05), with a significant difference only at transcriptional level. Significant main effects for the BPA treatment [F_(1,28)_ = 18.298, p<0.001], as well as for the gender [F_(1,28)_ = 9.273, p = 0.005] were found on P450arom mRNA levels; however, no main effect for treatment × gender interaction was observed [F_(1,28)_ = 0.0516, p = 0.822].

### Effects of BPA on Tph Isozymes

**Figure 4:**
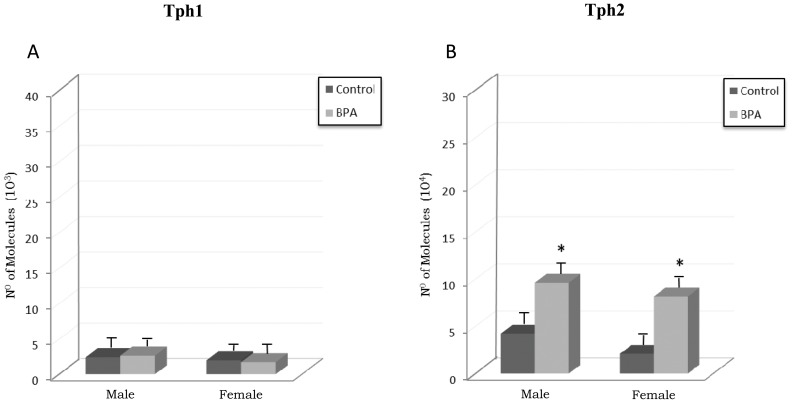
Effects of BPA on mRNA levels of tryptophan hydroxylase type 1 (Tph1) (panel A) and tryptophan hydroxylase type 2 (Tph2) (panel B) in prefrontal cortex of BPA-treated male and female rats and their respective controls. * at least p<0.05 vs. their controls.

Figure 4 depicts the mRNA levels of Tph1 (panel A) and Tph2 (panel B) in PFC of BPA-treated rats and their controls. After BPA treatment no significant differences in Tph1 mRNA levels were observed in males and females versus their respective controls. Statistical analyses revealed significant main effect for gender [F_(1,28)_ = 15.760, p<0.001] on Tph1 mRNA levels; however, no main effects for BPA treatment [F_(1,28)_ = 0.0155, p = 0.902] or treatment × gender interaction [F_(1,28)_ = 1.997, p = 0.169] were identified.

A significant increase in Tph2 mRNA levels (Fig. 4B) was observed in males and females versus their respective controls after BPA treatment (p<0.001). Significant main effects for the BPA treatment [F_(1,28)_ = 61.903, p<0.001], as well as for the gender [F_(1,28)_ = 6.814, p = 0.014] were found on Tph2 mRNA levels; however, no main effect for treatment × gender interaction was observed [F_(1,28)_ = 0.0825, p = 0.776].

**Figure 5:**
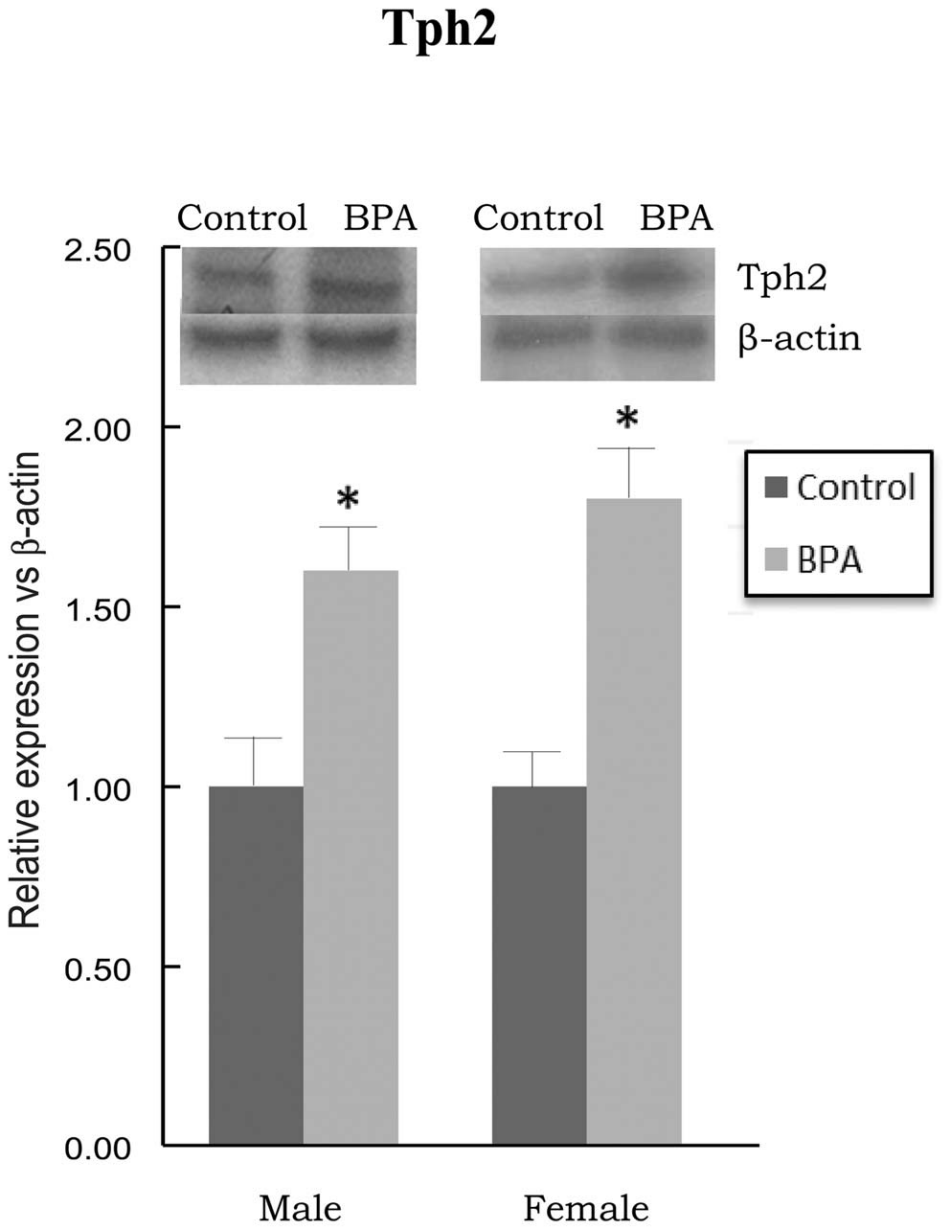
Effects of BPA on protein levels of tryptophan hydroxylase type 2 (Tph2) in prefrontal cortex of BPA-treated male and female rats and their respective controls. * at least p<0.05 vs. their controls.

A significant increase in Tph2 protein levels (Figure 5) was observed in males and females versus their respective controls after BPA treatment (p<0.05).

### Effects of BPA on Genes Involved in Neurotoxic Responses

**Figure 6:**
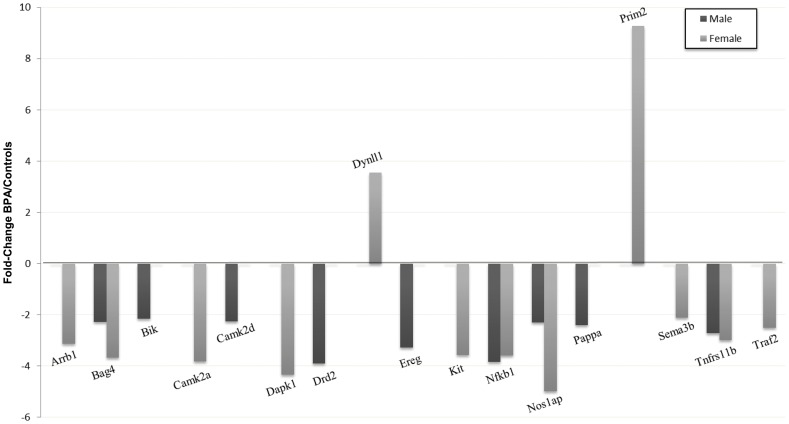
Effects of BPA on relative expression of genes involved in neurotoxic responses in prefrontal cortex of male and female rats. At least p<0.05 vs. their controls.

Table 2 depicts the 17 genes of the Rat Neurotoxicity PCR Array that were significantly modified by BPA treatment in PFC. 9 genes were down-regulated in BPA-treated males, whereas 10 genes were down-regulated and 2 genes were up-regulated in BPA-treated females (Figure 6).

**Table 2:** List of PCR array genes changed by BPA treatment in prefrontal cortex of rats.

Gene ID	Gene	Full name
25387	*Arrb1*	arrestin, beta 1
361167	*Bag4*	BCL2-associated athanogene 4
114496	*Bik*	BCL2-interacting killer (apoptosis-inducing)
25400	*Camk2a*	calcium/calmodulin-dependent protein kinase II alpha
24246	*Camk2d*	calcium/calmodulin-dependent protein kinase II delta
306722	*Dapk1*	death associated protein kinase 1
24318	*Drd2*	dopamine receptor D2
58945	*Dynll1*	dynein light chain LC8-type 1
59325	*Ereg*	epiregulin
64030	*Kit*	v-kit Hardy-Zuckerman 4 feline sarcoma viral oncogene homolog
81736	*Nfkb1*	nuclear factor of kappa light polypeptide gene enhancer in B-cells 1
192363	*Nos1ap*	nitric oxide synthase 1 (neuronal) adaptor protein
313262	*Pappa*	pregnancy-associated plasma protein A
5558 (Hs)	*Prim2a*	primase, DNA, polypeptide 2 (58 kDa)
363142	*Sema3b*	sema domain, immunoglobulin domain (Ig), short basic domain, secreted, (semaphorin) 3B
25341	*Tnfrsf11b*	tumor necrosis factor receptor superfamily, member 11b
311786	*Traf2*	Tnf receptor-associated factor 2

## Discussion

The results of this study indicate that adult exposure to BPA, even at short-term and at a dose considered safe, produces alterations in the expression of key genes for the rat PFC function in a sex-specific manner.

We report for the first time, at least to our best knowledge, that BPA administration to adult rats results in a decrease of 5α-R1 expression in PFC of female but not in male rats. However, neither 5α-R2 nor 5α-R3 were modified by BPA at the dose assayed. These data are very interesting because 5α-R1 is the main isozyme implicated in the biosynthesis of 3α,5α-NS such as AlloP [13], with higher levels found in females than in males [29]. Given that variations in the levels of AlloP are involved in the vulnerability for mental and emotional pathology via GABA_A_-R [30], reduced brain levels of 5α-R1 and, consequently AlloP, may contribute to increased susceptibility to these disorders in females. Thus, mood changes during the menstrual cycle, postpartum, major depression and epilepsy are pathologies associated with low AlloP levels [31].

BPA increased P450arom expression mainly in male rat PFC. Previous studies carried out in other brain areas of animal exposed to BPA during early life stages have also showed increased P450arom levels [32], [33]. P450arom catalyzes the conversion of androgens to estrogens, which are able to reduce the synthesis of GABA [34] and GABA_A_-R subunits [17]. Therefore, this increase in local P450arom expression by BPA in males along with the decrease in 5α-R1 in females reinforces the idea that BPA may affect GABAergic neurotrasmission in the adult PFC of both male and female rats.

Besides GABA system, brain 5-HT neurotransmission also regulates PFC function and the deregulation of this neurotransmitter could also lead to neuropsychiatric disorders [35]. In this study, BPA-treated rats showed an increase in Tph2 expression, a key isozyme in central 5-HT transmission [24]. According to our results, it has been reported an increase of 5-HT [36], [37] and Tph2 [38] levels in rodent brain after BPA exposure. Given that estrogens can regulate 5-HT levels increasing Tph2 expression [39], BPA may affect Tph2 through P450arom induction. In view of our results, with BPA increasing in a greater manner Tph2 in females than in males and P450arom in males than in females, another molecular mechanism of BPA on Tph2 should be kept in mind.

In this study, we also identified additional target genes of BPA in PFC of adult rats using the PCR-array technology. Thus, we observed in female rats that BPA decreased the mRNA levels of *Arrb1*, a gene which encodes for a G-protein-coupled receptor adaptor protein implicated in protective signaling through group I metabotropic glutamate receptors (mGlu1a) [40]. A reduction in Arrb1 levels has been linked with the physiopathology of mood disorders (e.g., major depression) [41]. Therefore, BPA may contribute to an increased susceptibility to these diseases in the female by decreasing both Arrb1 and 5α-R1.

Interestingly, we found that BPA affects several synaptic plasticity and memory-related genes. Thus, BPA decreased the mRNA levels of *Camk2d* in male and *Camk2a* in female rats. The products of these genes are the δ and α chains, respectively, of the calcium/calmodulin-dependent protein kinase II (CaMKII). CaMKII is essential for memory consolidation and certain forms of synaptic plasticity such as long term potentiation (LTP) [42]. Since CaMKIIα is one of the major forms of CaMKII in brain [43], our results suggest that adult exposure to BPA might affect CaMKII function mainly in female. In this line, Viberg et al. [44] have found that neonatal exposure to BPA decreased CaMKII levels in cerebral cortex of adult female mice but not in males.

CaMKII is also required for calcium-mediated activation of nuclear factor-kappa-B (NF-κB), which is critical to host defense and has been implicated in long-term changes in synaptic plasticity [45]. In this study, BPA decreased in PFC of both sexes the mRNA levels of Nfkb1, a precursor of the p50 subunit of NF-κB. Consequently, NF-κB functions could be compromised. According to our results, other authors have reported that BPA inhibits activation of NF-κB in macrophages [46].

BPA decreased the mRNA levels of *Nos1ap*, an adaptor protein that link neuronal nitric oxide synthase (nNOS) to specific targets [47], mainly in female rats. nNOS has been implicated in modulating physiological functions such as learning, memory and neurogenesis [48]. We also observed that BPA increased the mRNA levels of *Dynll1*, an inhibitor protein of nNOS [49], only in females. Therefore, adult exposure to BPA might have sex-specific effects on the nitric oxide system, according to Martini et al. [50].

In this study we also observed a decrease in mRNA levels of *Kit* in female rats after BPA exposure. Low activity of Kit has been associated with impaired spatial learning and memory in adult rats [51].

Another important finding was that BPA administration to male rats decreased the mRNA levels of *Drd2*, which is crucial for PFC cognitive function [52], [53]. Interestingly, it has been described that BPA produces abnormal development of synaptic plasticity in the striatum of rats due, in part, to down-regulation of Drd2 function [54].In BPA-treated male rats we also observed a decrease in the mRNA levels of *Ereg*, a ligand of ErbB family of receptor tyrosine kinases. These receptors are involved in the regulation of GABAergic transmission in the adult PFC [55] and in synaptic plasticity [56].

In addition, in our BPA-treated male rats we found decreased mRNA levels of *Pappa*, a metalloproteinase which cleaves insulin-like growth factor binding protein 4 (IGFBP4), thus regulating local IGF bioavailability [57]. Interestingly, brain IGF regulates learning and memory [58] and has also a neuroprotective role after brain injury [59]. Although many studies have reported that BPA alters IGF signalling [60], here we show a new mechanism by which BPA may affect this system in the adult brain.

In BPA-treated female rats we observed a decrease in mRNA levels of Sema3b, which encodes for an important axon guidance protein [61].

Taken together, our findings clearly show that adult exposure to BPA alters genes implicated in mechanisms involved in synaptic plasticity and memory in PFC of the rat. Although, we also found that BPA produces changes in the transcription of other genes related with such important functions as cell survival (*Prim2a*) and cell death (*Bik, Tnfrsfl1b, Dapk1, Bag4 and Traf2*), which may also contribute to alter the physiological function of PFC.

## Conclusions

This study shows that short-term exposure to BPA in the adulthood, at the EPA’s current reference safe daily limit, causes alterations in the expression of genes involved in PFC physiopathology. These changes may affect important functions of this brain area, such as higher cognitive functions and normal emotional processing, and might contribute to increased susceptibility to several psychopathologies in the adulthood.

These findings represent a significant contribution to the limited knowledge on the neurotoxic effects of BPA exposure in the adulthood. They should be taken into consideration by the environmental health policies to encourage efforts in the process of risk assessment of this endocrine disrupter.
